# Diet for Pyorrhea Alveolaris

**Published:** 1903-10-15

**Authors:** J. Warren Achorn


### DIET FOR PYORRHEA ALVEOLARIS.

BY J. WARREN ACHORN, M.D.

If water were the only food required, other things being equal, every one's health should be equal to every other one's. But things never are equal, and so there is a decided difference between water and its effects and what this or that one eats or drinks; something makes this one fat and that one lean, this one plethoric and that one anemic, this one rheumatic and that one lithemic or asthmatic. Inheritances, habits, work, surroundings, temperament, play, food, and drink, all have to be considered in the sum of the things that finally get a well man out of order.

Most of us go on eating and drinking and doing things we learned to do unconsciously as children or acquired in some other thoughtless way without regard to the after-effects, never once seriously asking ourselves whether as individuals this is the right thing for us, until some fine morning disease drives us to consult *a doctor*, and we find we are in for it, with no better reason or excuse than this, that we have taken too much for granted.

*The Personal Equation in Eating.*—Many people eat seven things right and in the right way, but Heaven only knows what they will do when it comes to that eighth thing, that something they like in particular, that has six inches of "taste good" in it for them and twenty-three feet of stomachache afterward for solution. It may be this eighth thing alone that plays havoc with their digestion and metabolism. Controlling the indiscriminate use of some one food or class of foods will work wonders occasionally. One person may eat a thing objectionable for the average individual and never hear from it, because for him there is nothing indigestible about it; his neighbor, looking on, thinks he can do the same, not appreciating the individual factor in the equation, and so it goes until they are all at it.

It is all summed up in the expression, "Oh, that won't hurt you."

There was probably a time when a man could eat anything in sight, simply because he lived, like any other animal or vegetable, in the open air, and did not have much to chose from; his diet was sustaining, but a simple one. Now he is fenced in by stone buildings, he walks on stone streets, and looks out upon the world through a hole in the wall. Is it any wonder, in view of all the changes to which he has been subjected, in view of the innumerable things he is doing in a hurry, and the foods he is eating in a hurry, brought from all parts of the world and cooked in all sorts of ways, that his digestion fails, that he gets out of tune or out of proportion and finds himself troubled with some chronic ailment, the result of misfit food, waste in his system, or something worse? What one can eat with impunity at twenty is not necessarily the food that fits best at forty; with each decade the food that one eats would better be modified to satisfy special indications or changes incident to age and occupation. Would it not be a wise precaution for every one to consult his physician once a year at least, and let him say what if anything is wrong with the machine, the way it is run, or the food it is given. We all go to our tailor, our hatter, our cobbler and may I not say to our dentist (for I have an idea that people go more readily to the dentist than to the doctor) with fair regularity, and why not to the chief engineer of the body for inspection and a certificate good for six months or a year? Preventive medicine is the only true way to practice the art, but the world does not think so.

*The Physiological Balance.*—If a man's lung capacity is what it should be compared with his weight, and his lung capacity is not below the standard for his height, if his blood and urine are right under the microscope, and all his eliminative organs are acting normally, if his food and drink are sufficient to maintain his health and strength against his work and habits, if he is physically sound and at peace with himself, he is as near perfect as he can ever hope to be in this world. My definition for all this is "physiological balance," something all persons should strive to keep if they have it, or to attain by right life and living if they haven't it.

Chronic disorders, such as rheumatism, lithemia (American gout), real gout, dyspeptic asthma, certain kinds of headache, gastric hyperacidity, biliousness and acne, are recognized by physicians generally as due in part and in some instances entirely to disorders of digestion—the result of misfit foods or failure of elimination from whatever cause.

*Nutrition and Pyorrhea.*—It was while engaged in rectifying ills of this sort by dietetic treatment and other associated means when indicated that I incidentally treated pyorrhea alveolaris and succeeded in relieving some cases and curing others. I did this unconsciously in the beginning, the disease being a local disorder to my mind at that time.

Sometimes the case was one of obesity complicated by rheumatism; sometimes one of hyperacidity associated with headaches or constipation; sometimes lithemia or biliousness was the fundamental symptom, and sometimes there was no diathesis. The patient was out of tune mentally and had lost his balance physically from overwork or worry, or was handicapped by a small pair of lungs, or by a weak heart and small digestive organs, that in turn affected his elimination or nutrition; in a word, the machinery was not adequate to the mill in which it was set up; and here is where this idea of *balance* finds its greatest application.

If the case under consideration was one of the uric acid variety, as the majority of them were, I restricted the use of acids for a time, whether found in meat, fish, vegetables or condiments. When this diathesis was not present either in the family history or in the patient, I dieted according to the indications, always with a view to securing relief by gaining the coveted physiological balance.

*Predisposing and Substitute Foods.*—I am satisfied that meat and sugar in excess aggravate the group of disorders under discussion. How much this disease is conditioned upon the local effect of these and other foods I am unable to say. Curiously enough, the uric acid in nuts, even though they be identical, do not act in the same way when taken into the system. I have seen aches and pains follow the use of meat, and this was not the case when nuts were ingested.

The uric acid foods are the red meats—steak, roast beef, etc., and all viscera, such as liver, sweetbreads and kidneys. Among the liquids may be mentioned tea, coffee, cocoa, beers and soup made from meat stock. Mutton, lamb and veal are among the least objectionable of the red meats. The red meats that seem to be an exception to the rest are venison and rabbit. Wild animals, being much more active and having purer food and water to eat and drink than the stall-fed animals of commerce, are freer, I imagine, from waste products—from their own rheumatism if you please. At any rate, these meats do not act as injuriously as those that are stall-fed.

The substitutes for red meat are the white meat of chicken, turkey, capon, fresh fish (that is, they contain less uric acid), eggs, oatmeal, gluten maccaroni, peas, beans, nuts, milk and cheese. I mention peas, beans and nuts here, because they do not produce in my experience the same effect upon the system that the commercial red meats do. With some, red meat may be eaten once a day aside from fish. White meat and fish I rate about the same, except that white meat is the better digested. If there are "symptoms," vague aches that persist, I generally stop meats altogether, unless sufficient water is being taken to keep the system free.

The substitute drinks for tea, cocoa, coffee, etc., are the cereal coffees, water, milk, buttermilk and white wines in moderation.

The uric acid vegetables are peas, beans, and here I will mention nuts. Then there are those that contain oxalate of lime, which is akin to uric acid in its action on the system, asparagus, spinach, cabbage, rhubarb, tomatoes, carrots, string beans, celery and radishes. Vegetables containing these acids should have a restricted use, as may be determined by the condition of the patient and in part by examination of the urine.

The best sugars are pure maple syrup in season, pure honey for those who can eat it, sugar or milk and saccharin. When maple syrup can not be obtained, syrup made from the maple loaf is preferable to the granulated sugar. This choice is due to the difference in local effect, the maple syrup in my experience being less irritating to the stomach. Maltose and other western products of the kind are useful, but a taste for them has to be acquired. With the sorghum of the sugar cane I have had no professional experience. Sugar in measured quantities is a desirable food for children, with their activities and rapid digestion to be satisfied, coupled with the demand for a food that will afford quick energy and make ready fat; but for grown people its use in excess is unhealthful, a source of intestinal fermentation and of irritation throughout the system. Its free use and that of red meat in the class of disorders enumerated is certainly bad.

Fruits have a limited use much the same as have vegetables. The innocent-looking cultivated strawberry is by no means innocent. It is usually in a state of mild decay when eaten, but this is smothered under sugar and cream. Even wholesome strawberries of the cultivated sort work mischief. There is something in them that causes aches and pains pointing to systemic disturbance. Sweet grapes and uncooked pears eaten freely induce lassitude, nausea and consequent loss of appetite. All fruits would be better cooked and eaten without sugar, or only a dash of it. The safest fruits are apples, raw or cooked, baked pears, white grapes, oranges, cantaloupes, plums and peaches. Bananas baked in their jackets are rendered quite digestible. I may add here that the free use of vinegar, pickles and condiments is unwise.

For the rest, most fine-fibered fish are allowable, if fresh such as cod, haddock, flounder, the lean of shad and bluefish, raw oysters and plain lobster, perch, bass, or other lake or brook fish, baked or broiled, but never fried. Fried food is an abomination. The frying-pan landed here in 1492 and from that day until recent times it has ruled over every stomach and kitchen in the land. The people in New England are still in love with it, and in the Southern States, where the standard of cooking is measured by the darkey mind, it rules supreme. I am honestly of the opinion that fried food has caused as much misery, first and last, as alcohol.

Eggs are often eaten in too great quantity. On account of the fat and iron contained in the yolks they produce the condition known as biliousness. They are better eaten on alternate days of the week, soft-boiled or poached.

The use of breads and cereals depends upon whether the patient can digest them readily, is in need of carbohydrates, or should be denied them. That starches aggravate the diseases under consideration, by inducing intestinal fermentation when not properly cooked, or when eaten in too great a quantity, is true. The starch of loaf bread, potatoes, beans and sweet potatoes is seldom thoroughly cooked and is therefore open to suspicion. White bread as ordinarily toasted is made worse by the operation, if toasted and redried, the starch is converted. Rye, graham and stale white bread are the best of the home-made kinds. Rusk, zwieback, the crust of French bread, Pilot bread and Cestus phosphated crackers, can all be used to advantage at times. Beans baked in the ground are all right. Cereals as now prepared for the market are a wholesome class of foods. Butter is the best fat, and next to that olive oil or cream. Bacon fat is also useful eaten with dry toast. The amount of fat and the kind depend upon the needs of the patient and his ability to digest it. Fats are not acted on in the stomach. Desserts are a snare; an inducement for those who have eaten enough to eat more only to do them harm. If a patient has a poor appetite that needs tempting they are permissible, but an equally good way to nourish is by a fourth meal at bedtime—strained oatmeal gruel, hot milk with Vigor chocolate, an egg in milk or swallowed whole in Moxie.

*The Use of Water.*—The unintelligent use or the lack of water is often a cause of chronic ailments, due to disorders of digestion. The best time to drink liquids is an hour before breakfast, between meals, and on retiring. Not more than one or two glassess should be taken at meal times, and no other liquid should be drunk if soup be indulged in. I may say of soups that in small amount they are not objectionable. In the diseases under consideration soups made from meat stock are contra-indicated; they are simply so much urea. Milk and meat do not go well together, or milk as a beverage with vegetables. Milk goes with eggs, fish, fruit or cereals. It were better sipped during the meal. Tea and meat do not go together; the tannin in the tea hardens the meat fiber. Cereal coffees, water, or wine go with meats. Some people wash their food down with whatever they may be drinking. No liquid should be drunk until after the food in the mouth is swallowed.

The constitutional treatment of Riggs' disease from a dietetic point of view is not essentially different from that of other disorders due to food not suited to the individual, to diatheses, inheritances, and bad habits. I insist in addition only upon the physiological balance when lost, or there is an approach to losing it, for I am satisfied that there may be cases of pyorrhea alveolaris, as there are other unhealthy physical states without a name, that are due to want of balance and that alone. You all know better than I the percentage of cases that are of purely local origin, and the immediate causes for it; probably the larger proportion of all cases would be in this class. I only hope, if you have intractable cases, whether or not under the bane of some diathesis, you will resort, to help you out, to dietetic and hygienic treatment such as the indications suggest. Riggs'

disease as I see it is a wholesale problem in certain cases, and the cure depends upon nothing being overlooked that tends to aggravate the trouble and upon everything being done that will raise or lower the individual until he is at par with himself and his surroundings.—*Cosmos.*

